# Tailoring the Structural and Optical Properties of Cerium Oxide Nanoparticles Prepared by an Ecofriendly Green Route Using Plant Extracts

**DOI:** 10.3390/ijms25010681

**Published:** 2024-01-04

**Authors:** Nicusor Fifere, Rodinel Ardeleanu, Florica Doroftei, Marius Dobromir, Anton Airinei

**Affiliations:** 1 Petru Poni Institute of Macromolecular Chemistry, 41A, Grigore Ghica Voda Alley, 700487 Iasi, Romania; fifere.nicusor@icmpp.ro (N.F.); rodar@icmpp.ro (R.A.);; 2 Department of Exact and Natural Sciences, Institute of Interdisciplinary Research, Alexandru Ioan Cuza University of Iasi, 11 Carol I Blvd., 700506 Iasi, Romania; marius.dobromir@uaic.ro

**Keywords:** cerium oxide nanoparticles, green synthesis, structural analysis, optical properties, plant extract

## Abstract

The present study explores an environmentally friendly green approach to obtain cerium oxide nanoparticles via a biomediated route using *Mellisa officinalis* and *Hypericum perforatum* plant extracts as reducing agents. The as-prepared nanoparticles were studied for their structural and morphological characteristics using XRD diffractometry, scanning electron microscopy, Raman, fluorescence and electronic absorption spectra, and X-ray photoelectron spectroscopy (XPS). The XRD pattern has shown the centered fluorite crystal structure of cerium oxide nanoparticles with average crystallite size below 10 nm. These observations were in agreement with the STEM data. The cubic fluorite structure of the cerium oxide nanoparticles was confirmed by the vibrational mode around 462 cm^−1^ due to the Ce-08 unit. The optical band gap was estimated from UV-Vis reflectance spectra, which was found to decrease from 3.24 eV to 2.98 eV. A higher specific area was determined for the sample using *M. officinalis* aqueous extract. The EDX data indicated that only cerium and oxygen are present in the green synthesized nanoparticles.

## 1. Introduction

Synthesis of metal oxide nanoparticles with characteristic sizes, tailored structure and morphology is an important aspect in the development of novel nanomaterials with enhanced properties, manufacturing of new nanostructures and investigating their applications. Cerium oxide nanostructures were globally obtained due to their excellent properties with potential applications in energy conversion and storage, catalysis cosmetic product solar cells, mechanical polishing, automotive exhaust treatments, the biomedical field and gas sensor therapeutic agents [1,2,3,4,5,6,7,8,9,10,11,12]. The unique properties of cerium oxide derive from its stable fluorite-type structure and the coexistence of the two (Ce^3+^ and Ce^4+^) oxidation states which can be switched to each other depending on the local environmental conditions [13,14]. These mixed valence states of the cerium oxide nanoparticles can induce the absorption and release of oxygen atoms under oxidizing or reducing conditions, leading to the presence of the oxygen vacancies on the surface or inside of cerium oxide lattice, which could be very useful to improve the material characteristics in order to apply in industrial and environmental fields. In this way, the cerium oxide nanoparticles can provide antioxidant or pro-oxidant activity, 3+ oxidation states being responsible for the antioxidant properties [14,15,16]. The concentration of the oxygen vacancies can be monitored by the synthesis conditions and will influence the optical and catalytic properties of the cerium oxide nanoparticles. Also, the control of the Ce^3+^/Ce^4+^ surface ratio is very important in the direction of the biological activity and toxicity of the cerium oxide nanoparticles.

To obtain cerium oxide nanoparticles of various sizes, shapes, or morphology, several methods were adopted. Currently, the cerium oxide nanoparticles can be prepared by different chemical and physical techniques such as coprecipitation, sol-gel, hydrothermal, solvothermal, sonochemical, microwave, ball milling, spray pyrolysis, combustion and so on [3,9,12,17,18,19]. However, these traditional methods are time-consuming, utilize toxic compounds and require high energy and expensive equipment, determining adverse effects on the human health and environment. The bio-assisted synthesis of the cerium oxide nanoparticles which use low-cost, safe, ecofriendly and nontoxic sources such as plant extracts, fungi, yeasts, bacteria or algae can overcome the threshold of the above-mentioned methods [2,19,20,21]. The biogenic route to prepare metal oxide nanoparticles does not produce toxic byproducts, being less energy-consuming, with minimum environmental impact, and can be applied to large-scale production [2,11,22].

The plant extracts represent an available and inexpensive source to obtain metal oxide nanoparticles. The extract can be prepared from different parts of the plant, namely leaves, seeds, bark, flowers, roots or fruit pulp. The plant extracts contain a variety of phytochemicals such as flavonoids, phenolic compounds, terpenoids, carbohydrates, amino acids and proteins, which have an important role in the synthesis of nanoparticles [21,22,23]. These secondary metabolites from the plant extracts can serve both as capping/stabilizing and reducing agents in the formation of the cerium oxide nanoparticles with well-defined sizes and shapes [24,25]. The plant extracts in which the content of flavonoids or polyphenols is higher provide obviously smaller cerium oxide nanoparticles, making them suitable for biomedical applications due to their autocatalytic properties and low toxicity [21,22,26]. Taking into account the unique redox properties and oxygen exchange on the nanoparticle surface, the cerium oxide nanoparticles show promising applications as antimicrobial, anticancer, anti-inflammatory, antidiabetic or antifungal agents, bioscaffolds or drug delivery vehicles [21,27]. The morphology, Ce^3+^/Ce^4+^ surface ratio, oxygen vacancies, zeta potential and the nanoparticle microenvironment can modify the redox activity mechanism and the biomedical effects of the cerium oxide nanoparticles [28].

Recently, the environmentally friendly synthesis of the cerium oxide nanoparticles using *Origanum majorana* L., *Aquilegia pubiflora*, *Rheum turkestanicum*, *Dillenia indica*, *Datura metel* L., *Ocimum tenuifloreum*, *Coriandrum sativum* and *Morinda citrifolia* plant extracts has been described [6,19,22,26,29,30,31].

*Melissa officinalis* is a perennial herb which belongs to Lamiaceae family, being effective in the treatment of headaches, indigestion, insomnia or depression [32,33]. *Hypericum perforatum* is a popular medicinal plant widely utilized in traditional and conventional medicine recommended for gastrointestinal and bile diseases, diabetes, skin diseases, psychological disorders and so forth [34,35].

In the present work, *Melissa officinalis* (*M. officinalis*) and *Hypericum perforatum* (*H. perforatum*) plant extracts, medicinal plants which grow in Romania, were used for the first time to prepare cerium oxide nanoparticles via an ecofriendly and sustainable procedure. X-ray diffractometry (XRD), scanning electron microscopy (SEM), energy-dispersive X-ray spectroscopy (EDX), UV-Vis reflectance and Raman spectroscopy were employed to characterize the obtained cerium oxide nanoparticles. Further, the cerium oxide nanoparticles were evaluated for photoluminescent properties.

## 2. Results and Discussion

The aqueous plant extracts of *Mellisa officinalis* and *Hypericum perforatum* were used as reducing and stabilizing agents for obtaining the cerium oxide nanoparticles. The complex chemical composition of *M. officinalis* plant extract covers secondary metabolites such as polyphenolic compounds (p-coumaric, rosmarinic, chlorogenic, caffeic and ferulic, protocatechuic acids), flavonoids (quercetin, kaempferol, apigenin, luteolin and rhamnocitrin), terpenoids (geraniol, citral, linalool, β-caryophenylene and nerol), triterpenes (ursolic and oleanolic acids), sesquiterpenes and tannins [32,33]. The most important phytochemicals revealed in plant extracts of *H. perforatum* consists of naphthodianthrones (hypericin, isohypericin and pseudohypericin), phloroglucinols (hyperforin and adhyperforin), flavonoids (rutoside, quercetin, quercitrin and isoquercitrinkaempferol), flavones (apigenin and luteolin), biflavones (biapigenin and amentoflavone), tannins, phenolic acids, essential oils in small amounts and xanthones [34,35,36]. The extract composition can be influenced by different ecological factors depending on the plant location as well as by the plant development.

**Figure 1:**
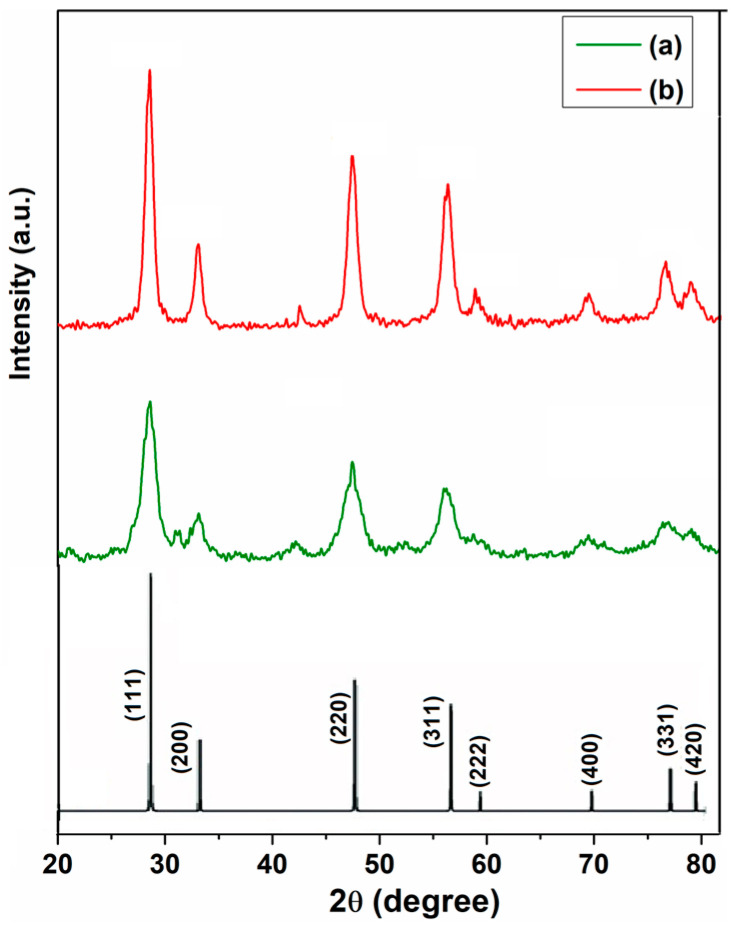
X-ray diffraction pattern of cerium oxide nanoparticles: (**a**) C-HP; (**b**) C-MO.

XRD data were applied to check the phase purity and the crystallinity of the cerium oxide nanoparticles prepared using plant extracts of *M. officinalis* and *H. perforatum*. Figure 1 depicts the XRD patterns of the cerium oxide nanoparticles C-MO and C-HP. The high-intensity diffraction peaks observed at 2θ, 28.52°, 33.12°, 47.49°, 56.34°, 59.07°, 69.66°, 76.96° and 79.25° (C-MO) correspond to the lattice planes (111), (200), (220), (311), (222), (400), (331) and (420), which are consistent with the face-centered fluorite structure of cerium oxide (JCPDS no. 34-0394) (Figure 1). The crystalline nature of the as-prepared cerium oxide nanoparticles is evident by intense diffraction peaks in the XRD pattern. 

The calculation of the crystallite size from X-ray diffraction measurements is based on the fact that they are small enough that the maxima of the diffraction pattern are broadened by an amount inversely proportional to the crystallite size [37,38]:

(1) $$\beta_{D}=\frac{K\lambda }{D\;\cos\theta }$$

Relation (1) is called the Scherrer equation, where $\beta_{D}$ represents the full width of half maxima (FWHM) of the most intense diffraction peak having the diffraction angle θ; k is known as the Scherrer constant, representing a shape factor, with a generally accepted value of 0.9; and λ denotes the X-ray wavelength.

The crystallite sizes, D, estimated with Relation (1) differ significantly, depending on the type of plant extract, having values of 5.05 nm for C-MO and 9.37 nm for the C-HP sample, respectively (Table 1).

The presence of imperfections and crystal distortion in the nanoparticles generates a strain in the crystal lattice that also leads to peak broadening, in addition to that caused by the size of the nanoparticle. According to the Williamson and Hall approximation, these two factors of peak broadening are independent of each other, and their contribution to the FWHM of the diffraction peak can be calculated by addition [39]:

(2) $$\beta =\beta_{D}+\beta_{\varepsilon }$$

where $\beta_{\varepsilon }$ represents the full width of half maxima due to strain *ε* of the network being directly proportional to it, according to the relationship:

(3) $$\beta_{\varepsilon }=4\varepsilon \;\tan\theta$$

**Figure 2:**
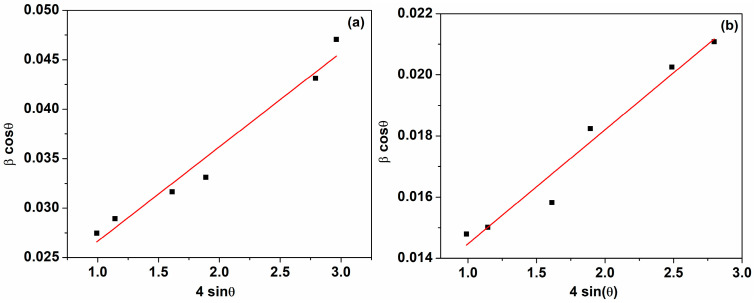
Williamson–Hall plots of the cerium oxide nanoparticles: (**a**) C-MO; (**b**) C-HP.

Using the last three relations, a linear equation can be obtained as follows [40]:

(4) $$\beta_{i}{\cos\theta }_{i}=\frac{0.9\;\lambda }{D}+4{\varepsilon \;\sin\theta }_{i}$$

where$\;\beta_{i}$ is the full width of half maxima of the i-th Bragg reflection positioned at $2\theta_{i}$, expressed in radians. From the linear representation of Equation (4) (Figure 2), the size of the nanoparticle, D, and the lattice strain, ε, can be obtained from the intercept on the y-axis and slope, respectively (Table 2). The dimensions of the nanoparticles calculated with the Williamson and Hall approximation (Equation (4)) are higher than those determined by the Scherrer equation (Equation (1)), as can be seen in Table 1.

It is well known that both methods of determining the size of nanoparticles are not exact, because they were obtained after some approximations. The values estimated by these methods represent averages that are more useful to analyze the size of the same nanoparticles prepared by different procedures [39]. Nevertheless, the C-MO sample has nanoparticle dimensions considerably lower than the C-HP sample, the difference between them (4.8 nm) being almost comparable to that obtained by the Scherrer equation (4.32 nm). The strain of the nanoparticles C-MO is more than double that of C-HP due to the smaller dimensions of the former, because the defects become much more significant with the decrease in the size of the nanoparticle [41].

In order to evaluate these results, we must take into account the fact that, generally, the crystallites have as subdivisions several domains with well-defined relationships between them. These domains are generated by two-dimensional defects, like twin boundaries or stacking faults, and are separated from each other by small differences in orientation of the crystal network. These assumptions were made taking into account the thermodynamic impossibility of obtaining a solid structure without imperfections. These differences in network orientation have angles of the order of units, generating the so-called small-angle grain boundaries, which scatter X-rays coherently [42]. Based on these considerations, it can be stated that *M. officinalis* extract, used to obtain the C-MO sample, introduces more grain boundaries which create smaller particles than those obtained with the *H. perforatum* extract, C-HP sample, decreasing the size of the coherent diffraction domain.

The lattice parameter, a, was calculated using Relation (5):

(5) $$a=\lambda \frac{{\left(h^{2}+k^{2}+l^{2}\right)}^{1/2}}{2\sin\theta }$$

where (hkl) are the Miller indices of the diffraction peak (111). The values of the average crystallite size and lattice parameter are given in Table 1. The values so obtained for the lattice parameter were 5.3765 Å for C-MO and 5.3986 Å for C-HP, respectively. These values are lower than that corresponding to the face-centered cubic cerium oxide (a_bulk_ = 5.4113 Å) (JCPDS card no. 34-394). It is observed that the lattice constant decreases with the particle size, although in most cases, an expansion of the lattice due to the size decrease can take place [43,44]. Also, it could be noticed that the value of the lattice parameter increases since the crystallite size increases (Table 1). This trend of decreasing lattice constant for the C-MO sample can be determined by the increased content of phytochemicals in *M. officinalis* extract leading to an efficient capping process, and crystallites with smaller sizes are formed. At the same time, the presence of capping agents contributes to a reduction in the surface relaxation of the forming nanoparticles leading to the smaller values of the nanoparticles. The decrease in the lattice constant can be caused by the existence of a microstrain on the nanoparticle surface [44,45,46].

Going beyond the X-ray data, other structural parameters such as lattice strain, dislocation density, specific surface area, X-ray density and stacking fault were determined. The dislocation density (δ) corresponding to the peak (111) was evaluated using the following relation [47,48,49]:

(6) $$\delta =\frac{15\beta \cos\theta }{4\mathrm{aD}}$$

As was expected, an inverse proportional relation was found between microstrain and average crystallite size, the reduction of the crystallite size determining a significant increase in the strain of C-MO (Table 2). The dislocation density indicates the defects per unit volume of the crystal structure. The value of the dislocation density was found to increase from 7.53 × 10^15^/m^2^ (C-HP) to 23.41 × 10^15^/m^2^ for the C-MO sample, suggesting that for this sample, the lattice defects increase (Table 1). Furthermore, the specific surface area can be evaluated from XRD data using the equation S = 6/Dρ [49,50], where S represents the specific surface area, D is the crystallite size and ρ is the cerium oxide density, respectively. It can be observed from Table 2 that the specific area of the C-MO sample was found to be 130.24 m^2^/g and 72.37 m^2^/g for the C-HP sample. In this case, C-MO presents a significantly higher specific area than the C-HP sample, and an increased surface area can determine high values of the surface energy, thus creating an increased number of active surface sites [50]. This fact can be related to the greater number of oxygen defects present in the C-MO sample. The values obtained for lattice strain and dislocation density agreed well with previously reported data [51]. The theoretical X-ray density (ρ_x_) for both samples can be estimated by the formula [52,53]:

(7) $$\rho_{X}=\frac{\mathrm{ZM}}{N_{A}V_{c}}$$

where Z denotes the number of atoms in the unit cell, M represents the molecular mass of the sample (172.115 g/mol), V_c_ refers to the unit cell volume and N_A_ is the Avogadro number. The calculated values of the X-ray density are inserted into Table 2. As can be seen, the X-ray density show close values for the two samples, and it increases as the unit cell volume decreases. The stacking fault (SF) is a planar defect which can appear during synthesis process, and this parameter can be determined using Relation (8) [48,51]:

(8) $$\mathrm{SF}=\frac{2\pi^{2}\beta }{45{\left(3\tan\theta \right)}^{1/2}}$$

The values of the stacking fault are given in Table 2. An evident decrease in strain, dislocation density and stacking fault was observed for the C-HP sample, improving its crystallinity.

**Figure 3:**
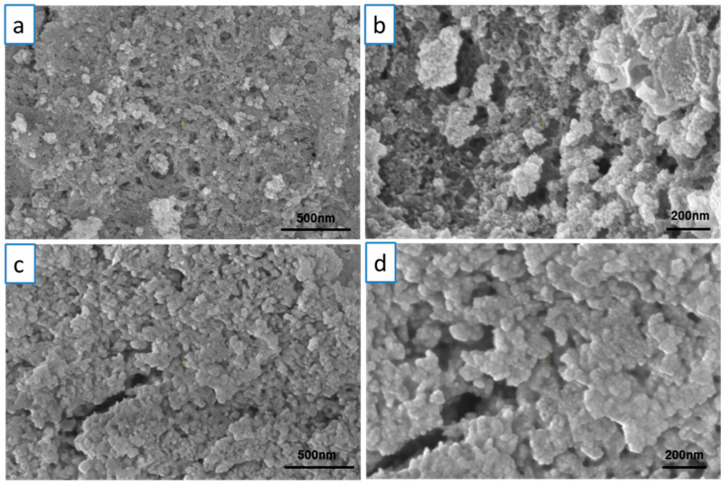
SEM morphology of cerium oxide synthetized nanoparticles at 50,000× and 100,000× magnification: (**a**,**b**) C-MO; (**c**,**d**) C-HP.

**Figure 4:**
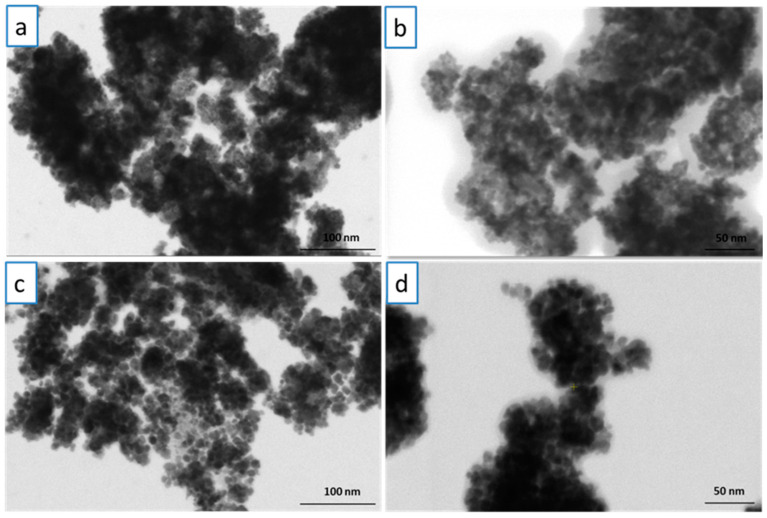
STEM-BF micrographs of synthetized C-MO and C-HP nanoparticles at different magnifications: (**a**) C-MO at 250,000×; (**b**) C-MO at 350,000×; (**c**) C-HP at 250,000×; (**d**) C-HP at 350,000×.

SEM micrographs obtained for the C-MO and C-HP samples are presented in Figure 3. In the case of the C-MO sample (Figure 3a,b), it can be observed that the nanoparticles have an approximately spherical shape with particles dimensions between 3.5 and 7 nm. C-HP nanoparticles show the same morphology, but a slight increase in the diameter of the particles can be observed. The sizes of the nanoparticles are in the range of 8–13 nm. Also, the cerium oxide nanoparticles prepared using aqueous extracts from *C. majus* and *V. album* presented a spherical shape as previously reported [54]. The results obtained from the morphological analysis of the two samples indicate that the synthesized samples have the same spherical morphology, but the particle sizes are different. In Figure 4, the STEM-BF micrographs of the two analyzed samples, at two magnifications 250,000× (Figure 4a,c) and 350,000× (Figure 4b,d) are presented. From these micrographs, it can be seen that the samples are homogeneous in terms of size and shape, and the results correlate with SEM imaging.

**Figure 5:**
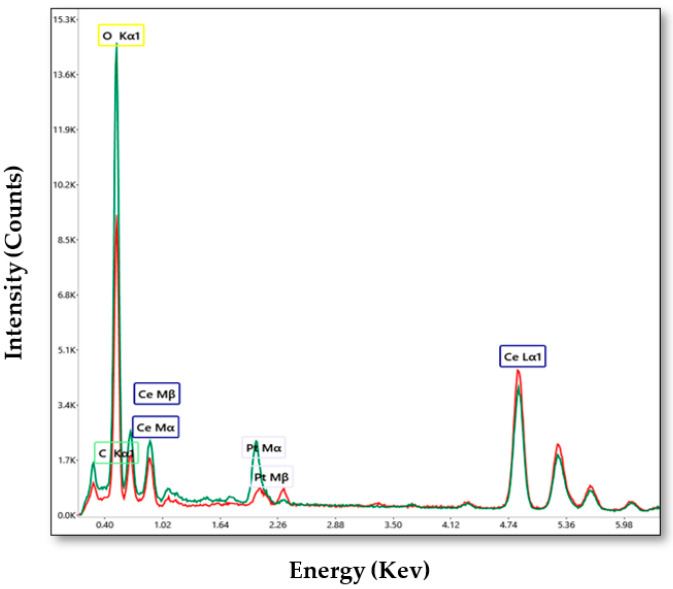
EDX spectra and chemical composition of the cerium oxide samples: C-H (red) and C-MO (green).

**Figure 6:**
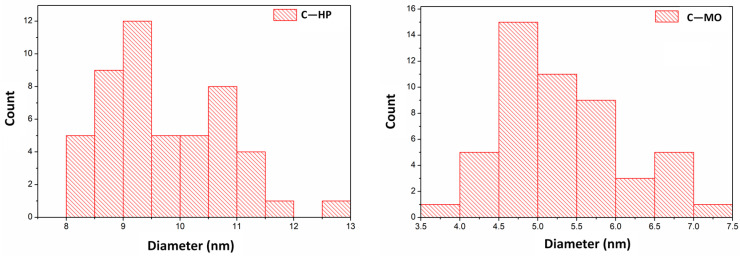
Particles size distribution of the cerium oxide samples determined from the STEM images.

EDAX (energy-dispersive spectroscopy analysis) was used to highlight the chemical composition of the samples. The EDX spectra of cerium oxide nanoparticles are shown in Figure 5. EDX spectra confirm the presence of Ce, O and C in both samples. In order to determine the average diameter of the obtained cerium oxide nanoparticles, 50 nanoparticles were measured for each sample using the Image J software (Version 1.46). The results are presented in the form of histograms in Figure 6. From the measurements, it was found that in the case of the C-MO sample, the average diameter of the particles is 5.3 nm, and in the case of the C-HP sample, the average diameter is 9.72 nm.

**Figure 7:**
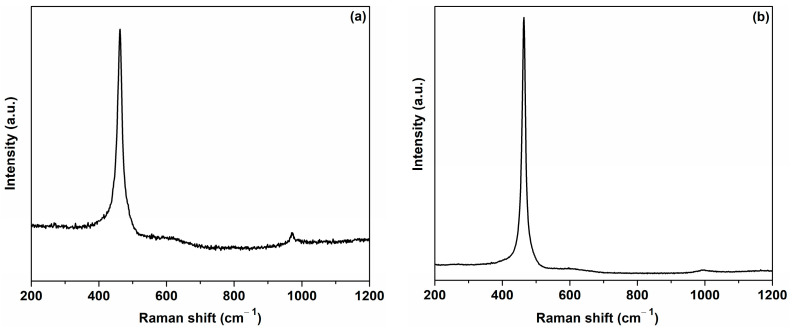
Raman spectra of cerium oxide nanoparticles: (**a**) C-MO; (**b**) C-HP.

The formation of the cubic structure in the cerium oxide nanoparticles was also proved by the Raman spectra. The strong intense Raman active mode located at 461.9 cm^−1^ for the C-MO sample and 463.5 cm^−1^ for the C-HP sample (Figure 7) corresponds to the F_2g_ symmetrical stretching vibration of the Ce-8O vibrational unit in a cerium oxide nanocrystal [46,55,56,57]. As seen from Figure 7, weak absorption bands of the cerium oxide nanoparticles prepared by the two plant extracts appeared at 273 and 600 cm^−1^, which were associated with second-order transverse acoustic (2TA) mode and to oxygen defect-induced mode taking into account the structural defects in the lattice, (D), respectively [58,59,60]. The main Raman band shifts to lower wavenumbers compared to the band of bulk ceria (466 cm^−1^) are due to the defects in the lattice and the variation in the phonon relaxation with the nanoparticle size [59]. The nanoparticle size can be calculated from Raman spectra using Relation (9) [61].

(9) $$\Gamma =5.48+98.4/D_{R}$$

where Γ denotes the width at half maximum of the F_2g_ mode (cm^−1^) line, and $D_{R}\;$is the particle size. Using the above relation, the following values for the grain size of the nanomaterials were obtained: 6.73 nm for C-MO and 12.66 nm for C-HP, respectively. These results are in good agreement with the values determined from XRD spectra (Table 1). The average distance separating two lattice defects (correlation length), L, can be estimated by Relation (10) [62,63,64]:

(10) $$L={\left\{{\left(\frac{\alpha }{2D_{R}}\right)}^{2}\left[{\left(D_{R}-2\alpha \right)}^{3}+4D_{R}{}^{2}\alpha \right]\right\}}^{1/3}$$

where α is the radius of the cerium oxide units (α = 0.34 nm) [62]. The defect concentration (N, cm^−3^) can be obtained from the correlation length, L, using the relation $N=\frac{3}{4\pi L^{3}}$ as a function of grain size [61,62,63,64]. From Table 1, it can be seen that the C-MO sample with the lowest particle size has a higher defect concentration.

**Figure 8:**
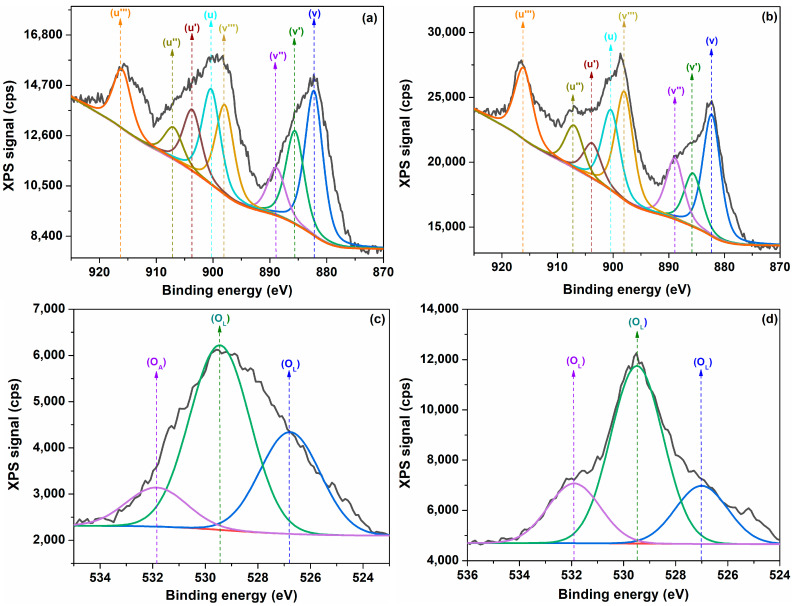
Ce 3d spectra: (**a**) C-MO; (**b**) C-HP; and O 1s spectra: (**c**) C-MO; (**d**) C-HP of cerium oxide nanoparticles from XPS measurements.

The oxidation states of cerium and oxygen in the plant-mediated samples were analyzed using X-ray photoelectron spectra (XPS). The Ce 3d core level spectra are given in Figure 8a,b for the cerium oxide nanoparticles. Cerium 3d peaks were observed in the binding energy range of 885 to 920 eV, assigned to trivalent Ce^3+^ and tetravalent Ce^4+^ [18]. As shown in Figure 8a, the Ce 3d core spectra consist of eight peaks arising from the spin-orbit splitting of the 3d_3/2_ and 3d_5/2_ states [65,66]. The peaks labeled as u‴ (916.28 eV), u″ (907.08 eV) and u (900.48 eV) belonging to 3d_3/2_/2 levels and the peaks v‴ (898.08 eV), v″ (888.88 eV) and v (882.48 eV) belonging to 3d_5/2_ levels correspond to the Ce^4+^ 3d states. The peaks labeled u’ (903.88 eV) referring to 3d_3/2_ level and v′ (885.68 eV) relating to 3d_5/2_ level are characteristic of Ce^3+^ 3d states in the crystal lattice [67,68]. The characteristic XPS peaks of Ce^3+^ and Ce^4+^ exist in both samples, being in good agreement with previously reported studies [18,67,69]. In this way, a mixture of cerium ions, namely Ce^3+^ and Ce^4+^, can be located on the nanoparticle surface. The presence of the Ce^3+^ state can determine more oxygen vacancies and defects. The separation binding energy between the u-u″, v-v″ and v-v′ peaks was found to be around 6.6 eV, 6.4 eV and 15.8 eV, respectively, being characteristic of Ce^4+^ in cerium oxide, which is in thorough agreement with the reported literature data [70,71]. Also, the binding energy difference between the v and v′ peaks is around 3.2 eV, and that between the u and v peaks is around 18.2 eV, close to the values mentioned for Ce^3+^ compounds [72]. The Ce 3d core level spectra of the cerium oxide nanoparticles prove evidently that the cerium ions exist in a Ce^3+^ and Ce^4+^ state mixture. The content of Ce^3+^ and Ce^4+^ ions can be estimated by the relations [7,18,66]:

(11) $$\left[{\mathrm{Ce}}^{3+}\right]=\frac{A_{{\mathrm{Ce}}^{3+}}}{A_{{\mathrm{Ce}}^{3+}}+A_{{\mathrm{Ce}}^{4+}}}100$$

(12) $$\left[{\mathrm{Ce}}^{3+}\right]=\frac{A_{{\mathrm{Ce}}^{4+}}}{A_{{\mathrm{Ce}}^{3+}}+A_{{\mathrm{Ce}}^{4+}}}100$$

where $A_{Ce^{3+}}$ and $A_{Ce^{4+}}\;$represent the integrated area of the C3d peaks for the (III) and (IV) oxidation states. The values of the concentration of Ce^3+^, Ce^4+^ ions and the Ce^3+^/Ce^4+^ ratio are listed in Table 3. According to Equations (11) and (12), the Ce^3+^/Ce^4^ ratio is higher for the C-HP sample (Table 3). The presence of Ce^3+^ ions can give rise to the appearance of Ce_2_O_3_ or oxygen vacancies in the cerium oxide lattice.

Figure 8c,d illustrate the O 1s core level spectra for the cerium samples giving information about the lattice oxygen, oxygen vacancies or absorbed oxygen. The peak at higher binding energies (531.88 eV (C-MO)) can be attributed to the adsorbed oxygen species on the cerium oxide surface (O_A_). The peaks appearing in the lower binding energy range (<530 eV) originated from lattice oxygen O^2−^, denoted as O_L_ [22,63,65,73]. Because the Ce^3+^ ions can be associated with Ce_2_O_3_ or oxygen vacancies, the oxygen content in cerium oxide can be estimated as the sum of the required oxygen to completely oxidize the cerium ions to give Ce_2_O_2_ and CeO_2_, respectively [65,74]. Since the stoichiometric ratio O/Ce for cerium oxide is 2 and for Ce_2_O_3_ is 1.5, the stoichiometric parameter, x, can be written as [74]

(13) $$x=3/2\left[{\mathrm{Ce}}^{3+}\right]+2\left[{\mathrm{Ce}}^{4+}\right]$$

Moreover, the actual stoichiometric ratio x′ can be evaluated from XPS data using the ratio of integrated areas of the O 1s and Ce 3d peaks according to the relation [18,67]:

(14) $$x^{\prime }=\frac{A_{O}}{A_{\mathrm{Ce}}}\cdot \frac{S_{\mathrm{Ce}}}{S_{O}}$$

(15) $$\Delta x=x-x^{\prime }$$

where A_O_ and A_Ce_ represent the XPS integrated areas of the O 1s and Ce 3d peaks, and S_Ce_ (7.399) and S_O_ (0.711) denote the sensitivity factors of cerium and oxygen atoms, respectively. The values of actual stoichiometry and the [Ce^3+^]/[Ce^4+^] ratio are given in Table 3. The stoichiometric ratio x′ is smaller than x for the two samples, suggesting that the Ce^3+^ ions contribute to the formation of the oxygen vacancies. From Table 3, it can be observed that the value of x′ of the C-HP sample is lower than of the C-MO sample, in which a great amount of oxygen vacancies was generated. We observed a significant increase of the Urbach energy (Table 1) for the C-HP sample due to the higher content of defect states in the gap corresponding to a high value of ∆x.

**Figure 9:**
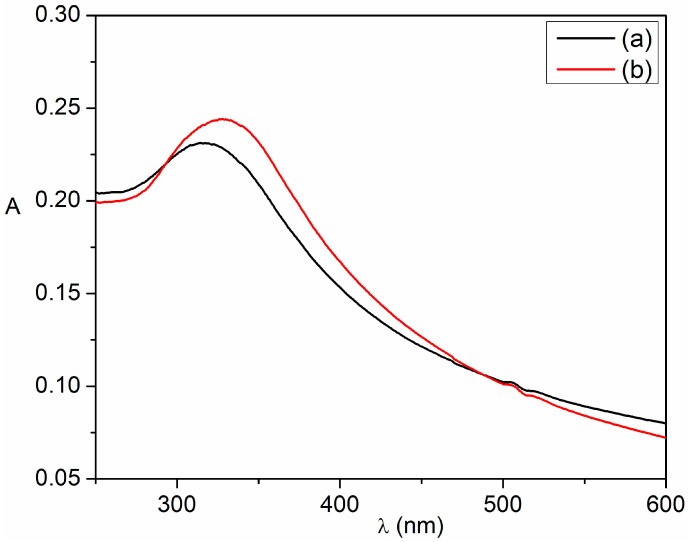
UV-Vis absorption spectra of the biogenic synthesized cerium oxide nanoparticles: (**a**) C-MO; (**b**) C-HP.

One of the key properties of nanostructures is their optical characteristics. The optical investigation of cerium oxide nanoparticles was carried out using electronic absorption spectroscopy. The electronic absorption spectra of the two samples of cerium oxide nanoparticles are illustrated in Figure 9. Both samples present an absorption band below 350 nm in the range 315–320 nm, which can be assigned to the charge transfer transition from O^−2^-2p to the Ce^4+^-4f orbitals in cerium oxide [51,75,76]. It is observed that the *H. perforatum*-extract-assisted cerium oxide nanoparticles exhibit an absorption band at 320 nm, but for the C-MO sample, the absorption maximum is shifted to shorter wavelengths. This blue shift can be due to the smaller particle prepared sample (C-MO) determined by a better capping effect of the cerium ions by the phytoconstituents contained in *H. perforatum* extract.

**Figure 10:**
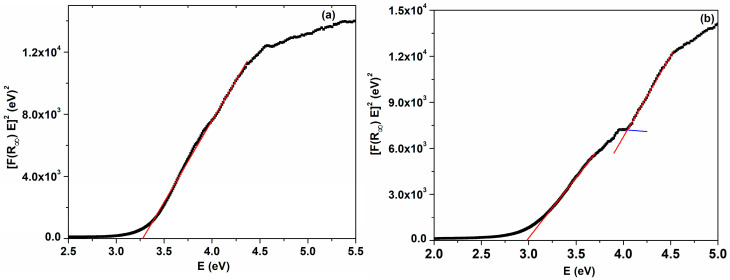

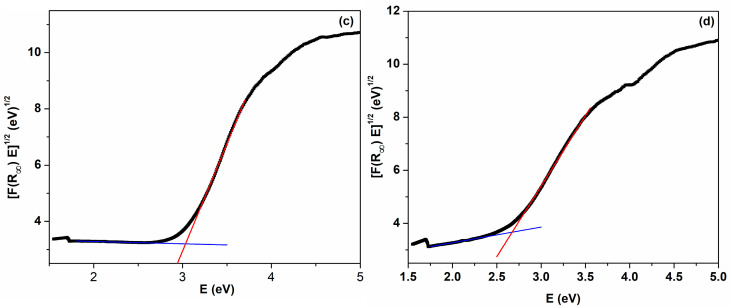
Plots of [F(R)hν]^n^ as a function of photon energy for cerium oxide nanoparticles: *n* = 1/2 (**a**) C-MO, (**b**) C-HP; *n* = 2 (**c**) C-MO, (**d**) C-HP. Red line refer to linear part of the plot, blue line refer to base line.

The optical band gap (E_g_) of the cerium oxide nanoparticles was calculated using the Tauc equation expressed as [77,78]:

(16) $$\alpha h\nu =A{\left(h\nu -E_{g}\right)}^{n}$$

where α represents the absorption coefficient, hν is the photon energy, A is a constant and *n* = 1/2 for a direct allowed transition and *n* = 2 for an indirect allowed transition. Using the UV-Vis diffuse reflectance data from thin films and the Kubelka–Munk function (17), Equation (16) can be as follows [79]:

(17) $$F\left(R_{\infty }\right)=\frac{{\left(1-R_{\infty }\right)}^{2}}{2R_{\infty }}$$

(18) $${\left[F\left(R_{\infty }\right)h\nu \right]}^{1/n}=A\left(h\nu -E_{g}\right)$$

where R_∞_ = R_sample_/R_standard_, $F\left(R_{\infty }\right)$ is the Kubelka–Munk function and R is the reflectance. Figure 10 illustrates the plots of ${\left[F\left(R_{\infty }\right)h\nu \right]}^{1/n}$ as a function of photon energy for the direct and indirect transitions. The direct ($E_{g}^{d}$) and indirect ($E_{g}^{i}$) values of band gap energy for cerium oxide nanoparticles can be extracted extrapolating the tangent drawn to the plot ${\left[F\left(R_{\infty }\right)h\nu \right]}^{1/n}$ vs. hν to ${\left[F\left(R_{\infty }\right)h\nu \right]}^{1/n}=0$ (Figure 10). From Figure 10b, it can be observed that the C-HP sample presents two energy gaps for the direct transition. The presence of two absorption edges in the band gap was also noticed for the cerium oxide nanoparticles obtained by coprecipitation and biogenic methods [7,54]. The obtained values of the direct band gap were found to be 3.27 eV for C-MO and 2.98 eV for C-HP, respectively. When observed, the direct and indirect band gap of the C-HP sample are smaller than the value corresponding to bulk cerium oxide ($E_{g}$ = 3.19 eV) [80,81]. It can be observed that the C-MO sample presents an increase in $E_{g}^{d1}$ value compared to the C-HP sample and bulk ceria. The decrease in nanoparticle size determines the increase in the energy difference between the lower energy level of the conduction band and the upper energy level of the valence band, and the $E_{g}^{d1}$ value for the C-MO sample becomes greater. These findings are in agreement with previous reports [7,18,65,82].

The decrease in the band gap energy is possibly due to the intrinsic defects (oxygen vacancies and Ce^3+^ ions) created in the cerium oxide lattice confirmed by XPS data for the C-HP sample. Also, the red shift in the band gap can be associated with higher content of oxygen vacancies and existence of Ce^3+^ on the nanoparticle surface. The reduction in the band gap energy could be a beneficial indicator for photocatalytic activity.

The optical band structure and the optical transitions are dependent on the width of the localized states near the absorption edge in the band gap which is known as the Urbach energy. The Urbach energy, $E_{U}$, correlates the tail width of the localized states with the crystal defects and lattice disorder, being a measure of the defect content between the conduction band and the valence band in the forbidden band gap. The relation between absorption coefficient, α, and Urbach band tail width can be written as [83,84]:

(19) $$\alpha =\alpha_{0}\exp\left(h\nu /E_{U}\right)$$

where $\alpha_{0}$ represents a constant. Taking into account that the absorption coefficient is proportional to $F\left(R_{\infty }\right)$, introducing the Kubelka–Munk function in Equation (19) and after linearization, Equation (20) becomes:

(20) $$\mathrm{lnF}\left(R_{\infty }\right)=\ln\beta +h\nu /E_{U}$$

where β is a constant.

**Figure 11:**
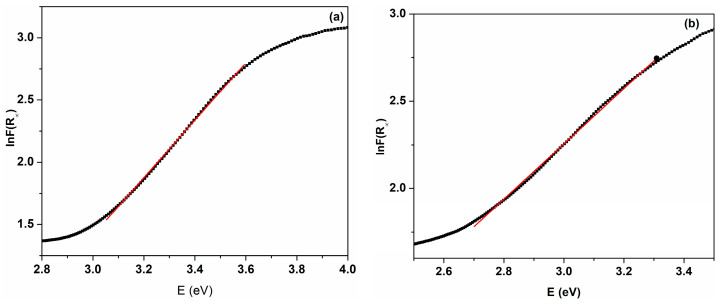
Urbach representations for cerium oxide nanoparticles: (**a**) C-MO; (**b**) C-HP. Red line refer to linear part of the plot.

Figure 11 displays the plots of $F\left(R_{\infty }\right)$ as a function of hν. The Urbach energy, $E_{U}$, was estimated from the reciprocal of the linearly fitted line slope. $E_{U}$ values are 433.37 and 631.31 meV for the C-MO and C-HP samples, respectively (Table 1). We note that a decrease in Urbach energy leads to a decrease in the structural defects for the C-MO sample, in agreement with XPS and XRD data. The decrease in the energy gap and the increase in the Urbach energy for the C-HP sample confirms the structural disorder of this sample and its higher level of defects.

**Figure 12:**
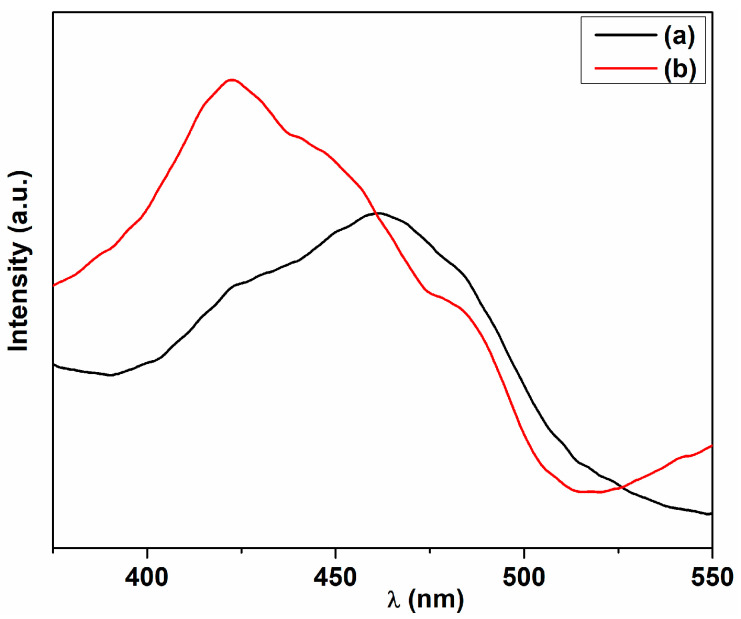
Photoluminescence spectra of cerium oxide nanoparticles: (**a**) C-MO; (**b**) C-HP.

The fluorescence spectra of the two ceria samples upon 300 nm wavelength excitation, at room temperature, in isopropanol suspension are given in Figure 12. As shown in Figure 12, the emission spectra of cerium oxide nanoparticles present a broad band character consisting of a strong violet band at 420–430 nm, a blue band around 460 nm and a blue-green band at 480 nm. These emission bands are in good agreement with data previously reported for plant-mediated prepared cerium oxide nanoparticles [46,85,86,87,88]. These emission bands can be assigned to different surface structural defects in cerium oxide nanoparticles including oxygen vacancies [86,87,89,90]. The violet emission bands around 420–430 nm can be due to the oxygen defect states existing between the Ce 4f level and O_2_ p band and may be related to the Ce^3+^ sites in the cerium oxide nanoparticles [46,86,91]. The blue and blue-green emissions can be associated with surface defects in the cerium oxide nanoparticles such as dislocations, impurities, ion vacancies, etc. [69,92].

A higher intensity of the emission in this region for the C-HP sample suggests the presence of oxygen vacancies that act as radiative recombination centers for electrons initially excited from the O 2p conduction band. These results are in good correlation with the XPS measurements. On the other hand, the intensity in the blue-green region is lower than that of the C-MO sample due to a more extensive presence of the structural defects that act as non-radiative recombination centers. The presence of such defects was highlighted in the results obtained from Urbach energy, the C-HP sample having the greatest value. The differences between the emission profiles of the two samples can be generated by different densities of defects determined by the plant components in the formation and stabilization of the nanoparticles.

## 3. Materials and Methods

### 3.1. Materials

Cerium (III) nitrate hexahydrate was supplied by Sigma Aldrich, Steinheim, Germany, and used without further purification. The solvents of spectrophotometric grade were bought from Sigma-Aldrich. The double-distilled water was utilized for the solution and preparation process.

### 3.2. Methods

X-ray diffraction (XRD) analysis was performed using a Bruker 18 Avance diffractometer (Bruker AXS, Karlsruhe, Germany) with CuKα radiation, operating at 40 kV and 40 mA. Data were collected between 10 and 90°. The crystallite size was estimated using the Scherrer equation, D = kλ/βcosθ, where λ represents the CuKα radiation wavelength (0.15406 nm), β is the peak full width at half maximum (FWHM), θ is the diffraction angle, in radians and k is a constant (0.90). A micro-Raman system (Renishaw in a Via Reflex) equipped with a 633 nm laser was employed to obtain Raman spectra of the cerium oxide nanoparticles. The optical properties of the dispersed samples in isopropanol were analyzed using UV-Vis absorption spectroscopy (SPECORD210Plus spectrometer, Analytik Jena, Germany) using 10 mm quartz cuvettes. Fluorescence spectra were studied using a PerkinElmer LS55 spectrometer in isopropanol with 10 mm path length quartz cells. X-ray photoelectron spectra (XPS) were taken on a Physical Electronic PHI-500 Versa Probe instrument using AlKα line (1486.6 eV) as exciting source. Morphological characteristics were investigated by scanning electron microscopy (SEM) on a Verios G4UC microscope (Thermo Scientific, Brno, Czech Republic) coupled with an energy-dispersive X-ray spectrometer (Octane Elect Super SDD detector, USA). STEM analysis was performed using a STEM3+ detector (bright-field mode) at an accelerating voltage of 30 kV. For this purpose, the cerium oxide nanoparticles were dispersed in water, then were deposited on carbon-coated grids and died to remove the solvent. For image analysis, ImageJ program was applied. All the materials of the analytical grade were procured from Sigma-Aldrich.

### 3.3. Preparation of Crude Extract of Melissa officinalis

*Melissa officinalis* was purchased from Plafar Company, Iasi, Romania. Firstly, the plant was washed thoroughly with running water followed by double-distilled water, and the material was dried under shade for 5 days. The dried plant was powdered using an electrical grinder. The extract was obtained placing 10 g of powdered plant in 100 mL of double-distilled water under mechanical stirring for 24 h at room temperature and then for 2 h at 50 °C. The suspension was cooled at room temperature and filtered using Fisher Whatmann-540 filter paper, and the extract was kept at 5 °C for further use.

### 3.4. Preparation of Cerium Oxide Nanoparticles

The cerium oxide nanoparticles were prepared using *M. officinalis* aqueous extract as a bioreducing and surface-modifying agent. In a typical procedure, 8.68 g of cerium nitrate hexahydrate was dissolved in 10 mL of bidistilled water, and the solution was stirred until a transparent solution was made. In this solution, 90 mL of *M. officinalis* extract was added dropwise and kept under magnetic stirring at 50 °C for 30 min. The color of the reaction mixture changed from dark brown to a white-brown precipitate, indicating the formation of cerium oxide nanoparticles. After depositing at 5 °C for 24 h, the collected precipitate was centrifuged (6000 rpm) for 45 min and washed several times with ethanol and dried at 50 °C for 3 h. Then, the sample was calcined at 600 °C for 5 h in air atmosphere to obtain the final product.

### 3.5. Preparation of Hypericum perforatum Extract

The flowering aerial parts of the *Hyperium perforatum* were washed in running water and then with bidistilled water to remove the dust particles. The collected *Herba hyperici* were shade-dried at room temperature. Thereafter, the material was ground into fine powder. For extract preparation, 10 g of plant powder was suspended in 100 mL of bidistilled water under continuous magnetic stirring at 25 °C for 24 h and then at 50 °C for 2 h. Subsequently, the mixture was filtered through Fisher Whatmann-540 filter paper, and the filtrate was stored at 5 °C.

### 3.6. Fabrication of Cerium Oxide Nanoparticles via H. perforatum Herb Extract

The cerium oxide nanoparticles were obtained by a green chemistry procedure using *H. perforatum* plant extract as a reducing agent. For green synthesis of cerium oxide nanoparticles, 8.68 g of cerium nitrate hexahydrate was introduced to 10 mL of bidistilled water. This solution was added dropwise into 90 mL of aqueous *H. perforatum* extract under stirring at 50 °C for 50 min. As the reaction mixture cooled down at room temperature, the precipitate was stored at 5 °C for 24 h. The obtained precipitate was subjected to centrifugation (6000 rpm) for 45 min, washed several times with bidistilled water and dried at 50 °C for 3 h. In order to obtain cerium oxide nanoparticles, the product was calcined in furnace at 600 °C for 5 h.

## 4. Conclusions

In summary, for the first time, cerium oxide nanoparticles were prepared via an environmentally friendly, inexpensive and nontoxic method using aqueous extracts of *M. officinalis* and *H. perforatum* as precursors. Fabrication of cerium oxide nanoparticles was confirmed by XRD, SEM, Raman, UV-Vis and fluorescence investigations. The crystalline nature of the cerium oxide nanoparticles was established by X-ray diffraction. The crystallite size was determined by the Scherrer method and Williamson–Hall plots. SEM images revealed that the nanoparticles are almost spherical in shape, having an average crystallite size of 5.05 nm (C-MO) and 9.37 nm (C-HP), respectively. Band gaps were estimated using Tauc plots, and their values are placed in the visible range. The Raman spectra of the cerium oxide nanoparticles indicated the presence of a Ce-O vibration mode. According to the fluorescence analysis, the different emission profiles of the samples could be due to the different content of defects depending on the phytochemical diversity in the plant extracts. The reduction in the band gap energy and the increase in the Urbach energy indicated the formation of the defects and oxygen vacancies in a high degree between the conduction and valence bands. These findings have confirmed that a simple method for production of cerium oxide nanoparticles is possible using some underemployed plants which act as a stabilizing agent in the synthesis process.

## Figures and Tables

**Table 1:** Structural parameters of the cerium oxide nanoparticles.

Sample	λ_max_	D_Scherrer_	a	ν	D_R_	E_g_^d1^	E_g_^d2^	E_g_^i^	E_U_	N
	(nm)	(nm)	(Å)	(cm^−1^)	(nm)	(eV)	(eV)	(eV)	(meV)	(cm^−3^)
C-MO	315	5.05	5.3765	461.88	6.73	3.27		3.04	433.37	1.31 × 10^21^
C-HP	320	9.37	5.3986	463.50	12.66	2.98	4.04	2.67	631.31	6.88 × 10^20^

**Table 2:** Structural characteristics of the cerium oxide nanoparticles.

Sample	D_W-H_	ε	δ	S	ρ_x_	SF
	(nm)		(m^−2^)	(m^2^/g)	(g/cm^3^)	
C-MO	8.1	0.0954	23.41 × 10^15^	130.24	7.36	0.0113
C-HP	12.9	0.0373	7.53 × 10^15^	72.37	7.26	0.0067

**Table 3:** XPS parameters of the cerium oxide nanoparticles.

Sample	[Ce^3+^]	[Ce^4+^]	[Ce^3+^]/[Ce^4+^]	x	x′	Δx
	(%)	(%)				
C-MO	24.79	75.21	0.33	1.88	1.69	0.19
C-HP	15.52	84.48	0.18	1.92	1.46	0.46

## Data Availability

Data present in this study are available upon reasonable request from the corresponding author.
